# Persistent Differences in Reported Inpatient Rehabilitation-Bed Capacity Across 27 European and OECD Countries

**DOI:** 10.3390/healthcare14203500

**Published:** 2026-10-18

**Authors:** Erman Gedikli, Emre Yilmaz, Doğukan Kurç

**Affiliations:** 1 Department of Health Management, School of Health Sciences, Istanbul Medipol University, Istanbul 34815, Türkiye; egedikli@medipol.edu.tr; 2 Department of Physiotherapy and Rehabilitation, School of Health Sciences, Istanbul Medipol University, Istanbul 34815, Türkiye; dkurc@medipol.edu.tr

**Keywords:** rehabilitation, hospital beds, capacity planning, club convergence, health systems, OECD

## Abstract

**Highlights:**

**What are the main findings?**

• Reported inpatient rehabilitation-bed capacity remained markedly different across the included countries, with no panel-wide convergence detected.
• Growth in reported bed capacity showed no consistent positive association with population ageing.

**What are the implications of the main findings?**

• Inpatient bed stocks should be interpreted alongside community services, workforce, and service use when assessing rehabilitation provision.
• Comparable reporting and population-based monitoring can support rehabilitation planning across different service models.

**Abstract:**

**Background/Objectives:** Reported inpatient rehabilitation-bed stocks vary widely across countries, but their trajectories and relationship with population ageing are less clear. We examined convergence and associations with ageing in 27 European and OECD countries during 2000–2023. **Methods:** We combined Eurostat and OECD data into a 504-country-year panel. Analyses included sigma- and beta-convergence, the Phillips–Sul log-t test and exploratory club clustering. Monte Carlo checks used the actual panel dimensions. Mean-group, common-correlated-effects mean-group, and Driscoll–Kraay models examined associations between bed-capacity growth and changes in older-population shares. **Results:** Reported capacity ranged from 2.4 to 193.8 beds per 100,000 population in 2023. Panel-wide convergence was rejected, while beta-convergence estimates remained imprecise. Dispersion increased descriptively in the balanced core, but country-bootstrap intervals for its change and trend included zero. Associations between capacity growth and ageing showed no consistent positive pattern. Simulation results distinguished panel-wide testing from less stable club membership in short panels. **Conclusions:** Reported inpatient rehabilitation-bed capacity followed heterogeneous trajectories across the included countries. Population ageing alone did not explain capacity growth. These findings concern one component of rehabilitation provision and support joint monitoring of inpatient capacity, community services, workforce, and service use.

## 1. Introduction

Rehabilitation is a central health-system response to conditions that affect functioning. Cieza and colleagues estimated that 2.41 billion people worldwide have conditions that could benefit from rehabilitation [1], while subsequent Global Burden of Disease analyses describe substantial musculoskeletal and neurological burdens [2,3,4]. The World Health Organization’s Rehabilitation 2030 initiative calls for rehabilitation to be integrated into health-system planning [5,6]. Documented unmet needs [7] and population ageing, with its implications for health-care demands [8,9], make the distribution of rehabilitation resources an important planning question.

Rehabilitation is delivered through inpatient, outpatient, community, home-based, and remote services. Seijas and colleagues’ international stakeholder survey documented variation across these service models [10], and Winitsky and colleagues’ comparative review described differences in delivery and financing across 14 countries [11]. Hospital-bed planning methods [12,13] and international resource indicators [14] provide useful tools for examining inpatient capacity. The joint Eurostat-OECD-WHO collection reports beds designated for rehabilitative care under the HC.2 function [15,16]. This indicator identifies a specific structural resource; differences in service organization and reporting mean that it does not measure total rehabilitation provision.

Convergence analysis asks whether these reported bed stocks become more similar over time. Sigma-convergence is concerned with declining dispersion, beta-convergence is concerned with whether countries starting at lower levels grow faster [17], and club approaches allow distinct groups of trajectories [18,19,20]. Applications to health expenditure have reported varying degrees of convergence and clustering [21,22,23]. Harikrishnan and Hiremath also applied convergence methods to the provision of health centers [24]. Against this background, the specific contribution of the present study is to examine the long-run trajectories of reported inpatient rehabilitation beds, including age-denominator measures and their association with demographic change.

A targeted literature update on 30 September 2026 combined rehabilitation-bed and hospital-bed terms with convergence, Phillips–Sul, and club-clustering terms, and examined the cited resource and expenditure literature. It identified related health-resource applications but no matching application to the rehabilitative-care bed series in the retrieved literature. This scoped search motivates the present question without establishing an exhaustive priority claim.

We examined three study questions within the included country panel:

**H1.**  *Did cross-country dispersion in reported inpatient rehabilitation-bed capacity narrow or persist over time?*

**H2.**  *Was there evidence of catch-up growth or panel-wide convergence, and what exploratory groups of trajectories were identified?*

**H3.**  *Was growth in reported inpatient rehabilitation-bed capacity consistently associated with changes in the population share aged 80 years or older?*

The analysis covers 2000–2023 and separates the observed trajectories of this resource from inferences about rehabilitation need or the adequacy of national planning.

## 2. Materials and Methods

This longitudinal ecological study used country-year observations of reported inpatient rehabilitation-bed capacity in selected European and OECD countries. It analyzed publicly available aggregate data and required no individual participant recruitment or consent. Statistical terminology is defined in the Statistical Terms table.

The outcome was the reported stock of hospital beds designated for rehabilitative care: beds maintained, staffed, and available for patients whose principal intent of care is rehabilitation (HC.2) [15,16]. Eurostat was the primary source and OECD data supplied complementary observations, including non-European countries [25]. Both collections use the joint Eurostat–OECD–WHO questionnaire. Across 416 overlapping country-years, the correlation was 0.998 and the mean absolute difference was 1.97 beds per 100,000 population. The combined panel preferred Eurostat where both sources reported and used OECD otherwise: 27 countries, 504 country-years, and 426 Eurostat-sourced and 78 OECD-sourced observations. Total hospital-bed series were used to derive the rehabilitation share of hospital beds.

The original extraction used a fixed mapping of 31 European countries together with countries represented in the OECD source archive, yielding 50 candidate countries. Twenty-seven had positive rehabilitation-bed observations during 2000–2023 and entered the panel; 23 had no target observations in that extraction. Six additional countries represented in the Eurostat archive were outside the original mapping, including Moldova, North Macedonia, and Serbia, which had positive rehabilitation-bed observations. These three countries were not added retrospectively, so that all reported analyses refer to the same 27-country analytical sample; their omission is acknowledged as a limitation of the study (Section 4). Supplementary Table S1 identifies every archived-source country, its inclusion status, observed coverage, and reason for exclusion. Missing target observations are not interpreted as zero beds or evidence of absent rehabilitation services. The included countries therefore do not provide complete coverage of Europe or the OECD.

Population shares aged 80+ and 65+ years came from Eurostat for European countries and the World Bank for Canada, Colombia, Israel, Korea, and the United States; GDP per capita at purchasing-power parity came from the World Bank [26]. Age-denominator measures express beds per 1000 older inhabitants descriptively and per 100,000 in logarithmic analyses. These denominators indicate demographic context rather than measured rehabilitation need, which also depends on disability, disease burden, service use, and delivery models.

### 2.1. Analysis Panels

Analyses requiring constant country composition used a balanced core of 14 countries observed throughout 2000–2023 and an extended core of 21 countries observed throughout 2010–2023. Neither core contained interpolated observations. The balanced core comprised Austria, Belgium, Canada, Switzerland, Czechia, Germany, Spain, Finland, Hungary, Israel, Italy, Lithuania, Portugal, and Slovakia. The extended core additionally included Bulgaria, Colombia, Croatia, Ireland, Iceland, Poland, and Romania. Regression analyses used the available observations in the 27-country combined panel. Capacity measures entered in natural logarithms. The construction rules required observed endpoints, at least 22 of 24 years for the balanced core or 12 of 14 for the extended core, and no internal gap longer than two years. The countries retained under these rules in fact had complete series; so, no interpolation was applied.

#### 2.1.1. Diagnostics

We report the Pesaran CD test for cross-sectional dependence [27], the CIPS panel unit-root test [28], and the Pesaran–Yamagata slope-homogeneity test [29] for the relevant specifications (Table A1).

#### 2.1.2. Sigma-Convergence

For each year, we calculated the cross-country standard deviation of log capacity on the balanced and extended cores for total-population and older-population denominators. The original 95% intervals used 2000 bootstrap resamples of complete country trajectories, preserving each selected country across all years. We estimated linear time trends by OLS with Newey–West standard errors (three lags) and reported the final-minus-initial-year difference. These procedures quantify different sources of uncertainty: the HAC trend test concerns temporal variation for the observed country set, whereas the country bootstrap assesses sensitivity to country composition. An additional 5000-resample analysis calculated both the trend and endpoint difference from each same resampled panel (Supplementary Table S2).

#### 2.1.3. Beta-Convergence

Following the growth-regression tradition [17], annualized log-capacity growth over non-overlapping five-year windows (2000–2005, 2005–2010, 2010–2015, 2015–2020, 2020–2023) was regressed on the initial log level with window fixed effects and standard errors clustered by country, unconditionally and conditional on the annualized change in the 80+ share and initial log GDP per capita; a long-run cross-section (2000–2023, balanced core) complements the panel estimates.

#### 2.1.4. Phillips–Sul Log-t Test and Club Clustering

We used the Phillips–Sul log-t test and sequential club algorithm [19,20]. Natural-log series were smoothed with a Hodrick–Prescott filter (λ = 400) [30]. Relative transition paths *h_it_* divide each smoothed log series by its cross-country mean, and *H_t_* denotes the corresponding cross-country transition variance. The regression $\log\left(\frac{H_{1}}{\mathrm{Hₜ}}\right)-\;2\cdot \log\left(\log t\right)=\;a\;+\;b\cdot \log\left(t\right)+u_{t}$ used the final 70% of observations with Bartlett-kernel HAC standard errors; *t* < −1.65 rejected panel-wide convergence at the nominal one-sided 5% level. After core formation, the sieve examined every country outside that core, including countries bypassed during core search. The remaining group was tested before recursive clustering, and adjacent qualifying clubs were merged. Club memberships were interpreted as exploratory classifications of the observed trajectories.

Monte Carlo validation generated 2000 panels per scenario at N = 14/T = 24, N = 21/T = 14, and N = 14/T = 20, matching the balanced, extended and pre-COVID-19 analyses. Scenarios represented convergence to one limit, two known clubs, random-walk divergence, and slower transitions from dispersed initial conditions. Empirical rejection and exact membership-recovery rates, with binomial intervals, are reported in Table A1 and Supplementary Table S3. Exact two-club recovery was 99.95% at 14 × 24, 90.40% at 21 × 14, and 98.15% at 14 × 20. These checks distinguish scenario-specific operating performance from a general accuracy claim.

Associations with population ageing were estimated as Δln(*beds*)_it_ = *α*_i_ + *β*·Δ*s*80_i,t−1_ + *ε*_it_, where Δs80 is the change in the population share aged 80+ years and the dependent variable is annual log growth in reported beds per 100,000 total population. We used static mean-group estimates from country-specific OLS regressions [31], common-correlated-effects mean-group estimates (CCEMG) augmented with cross-country averages [32], and pooled country fixed effects with Driscoll–Kraay standard errors [33]. Variants used contemporaneous change, distributed lags, the share aged 65+, and a long-difference cross-section. These models test associations with demographic change, not equivalence to zero, or the adequacy of need-based planning.

Sensitivity analyses examined the pre-COVID-19 period, the rehabilitation share of total hospital beds, the population aged 65+ denominator, subperiods, and leave-one-country-out samples. These checks address analytic specification and country influence; they do not remove differences in national classification or service organization.

The original analyses used Python 3.13.14 with pandas 3.0.3, NumPy 2.5.1, SciPy 1.18.0, and statsmodels 0.14.6. The revision reproduced the supplied results from the archived panels, checked the club-selection steps against the published algorithm, and repeated simulation validation at the actual dimensions. An independent implementation in R 4.6.1 originally reproduced the log-t slopes, beta regression, dependence and unit-root diagnostics, and sigma trend (Table A4). Differences in HAC small-sample corrections explain the reported differences between Python and R log-t statistics.

### 2.2. Availability of Data and Materials

All data are publicly available: Eurostat (https://ec.europa.eu/eurostat), the OECD Health Statistics data explorer (https://data-explorer.oecd.org), and the World Bank Open Data API (https://data.worldbank.org). The full reproducible pipeline-Python and R code, every raw API response, the assembled panels and all outputs-is provided with the submission and will be deposited in a public repository upon acceptance.

### 2.3. Ethics Approval and Consent to Participate

Not applicable. This study analyzed publicly available, aggregated country-level data only; no human participants were involved.

### 2.4. Use of Artificial Intelligence

A large language model (Claude 5.5, Anthropic) was used, under the authors’ direction and supervision, solely to assist with language translation. The authors verified all data, analyses, results, and references, and take full responsibility for the content of this work.

## 3. Results

**Figure 1:**
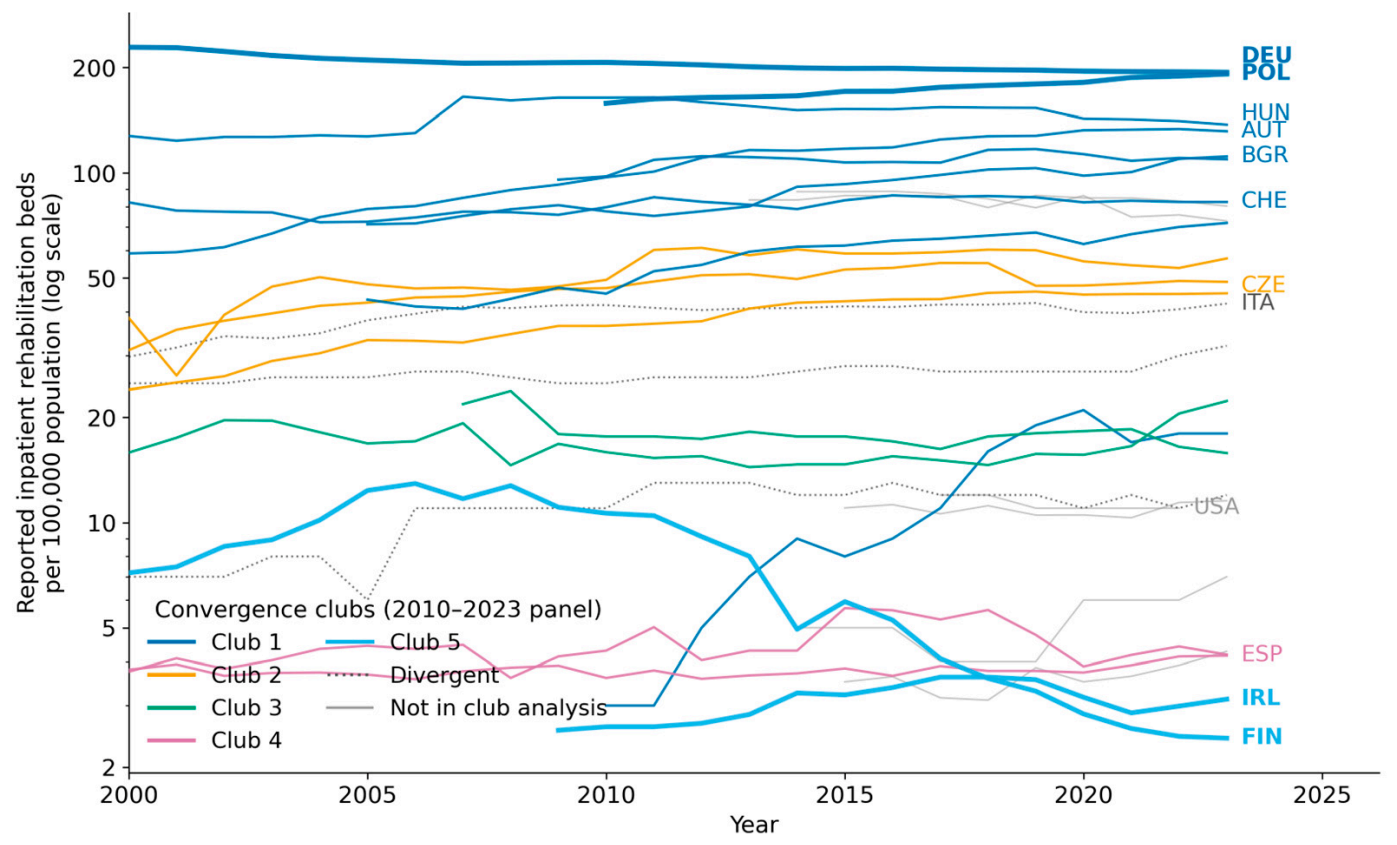
Reported inpatient rehabilitation-bed trajectories in the included countries, 2000–2023.

Reported inpatient rehabilitation-bed capacity in 2023 ranged from 193.8 beds per 100,000 population in Germany and 191.7 in Poland to 4.2 in Spain, 3.1 in Ireland, and 2.4 in Finland (Table 1; Figure 1). The rehabilitation share of all hospital beds also varied widely. These are comparisons of the reported inpatient category and do not rank total rehabilitation provision.

Each line represents one country’s reported rehabilitative-care beds per 100,000 population on a logarithmic scale. Colors indicate exploratory clubs from the corrected Phillips–Sul algorithm in the 21-country 2010–2023 panel; dotted dark-grey lines identify ungrouped divergent trajectories and light-grey lines countries outside that panel. Germany, Poland, Finland, and Ireland are shown with thicker lines. Short-panel club assignments should be read with the simulation results in Table A1.

Expressing capacity relative to older populations did not eliminate these differences. In 2023, Germany reported 26.9 beds per 1000 people aged 80+ years, compared with 0.41 in Finland. The cross-country correlation between beds per total population and the 80+ years share was 0.150 (*p* = 0.466). This descriptive comparison does not identify unmet needs or account for rehabilitation delivered outside the inpatient category.

Log capacity levels are strongly cross-sectionally dependent (CD = 7.66, *p* < 0.001, mean |ρ| = 0.498 in the union panel) and behave as unit-root processes (CIPS −1.88 against a 5% critical value of −2.39; first differences clearly stationary, −3.44), as expected for slowly evolving infrastructure stocks (Table 2). Growth rates, by contrast, show only weak dependence (CD = 0.97, *p* = 0.331), and the Pesaran–Yamagata test does not reject slope homogeneity for the responsiveness specification (_adjusted_ Δ = 1.20, *p* = 0.230). These diagnostics motivate the design: convergence methods built for nonstationary, transitionally heterogeneous panels (log-t), HAC/clustered inference throughout, and a CSD-robust estimator (CCEMG) wherever common shocks could contaminate the responsiveness panel.

**Figure 2:**
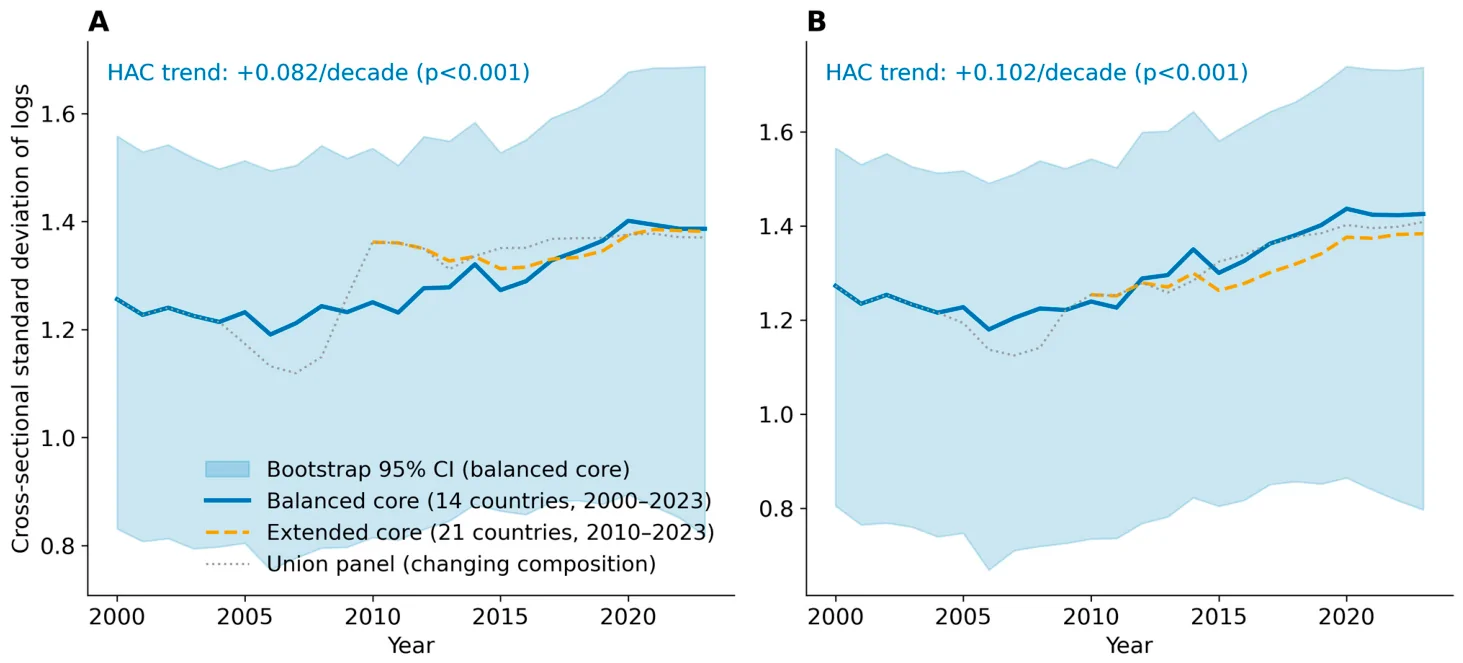
Cross-country dispersion of reported inpatient rehabilitation-bed capacity.

In the balanced core, the standard deviation of log capacity rose descriptively from 1.256 in 2000 to 1.387 in 2023 (Table 3; Figure 2). The temporal trend was +0.082 per decade (HAC *p* < 0.001), while the original country-bootstrap interval for the endpoint change included zero: +0.130 [−0.156, +0.423]. The additional joint resampling analysis also yielded a country-bootstrap trend interval spanning zero, from −0.047 to +0.243 per decade. Thus, the observed upward temporal trend was sensitive to country-composition uncertainty. Dispersion did not show a consistent narrowing pattern across specifications, but these results do not establish a precisely estimated rate of divergence across a wider population of countries (Supplementary Table S2).

Figure 2A uses beds per 100,000 total population; Figure 2B uses beds per 100,000 people aged 80+. Solid lines show the balanced 14-country core and shaded bands its original 2000-resample country-bootstrap pointwise intervals. Dashed lines show the 21-country extended core; dotted lines show the combined panel with changing composition. Annotations report conditional HAC time-trend tests. Country-bootstrap intervals for both trends and endpoint changes are reported separately in Supplementary Table S2.

The beta-convergence analyses did not detect consistent catch-up growth (Table 4). The unconditional five-year-window estimate was −0.0042 (95% CI −0.0144 to +0.0061; *p* = 0.423), with similar uncertainty after conditioning on demographic change and initial income. The balanced-core, age-denominator, and long-difference specifications likewise had intervals spanning zero. These estimates remain compatible with both modest convergence and divergence; they do not establish equivalence to no catch-up.

The Phillips–Sul test rejected panel-wide convergence in the balanced and extended cores (Table 5). For raw capacity, the balanced-core log-t statistic was −47.26 and the extended-core statistic was −70.42 against the nominal threshold of −1.65. The conclusion was unchanged across the reported trimming, smoothing, and leave-one-country-out checks (Table A3). Short-panel simulation results support distinguishing this panel-wide test from the more demanding task of assigning countries to individual clubs.

**Figure 3:**
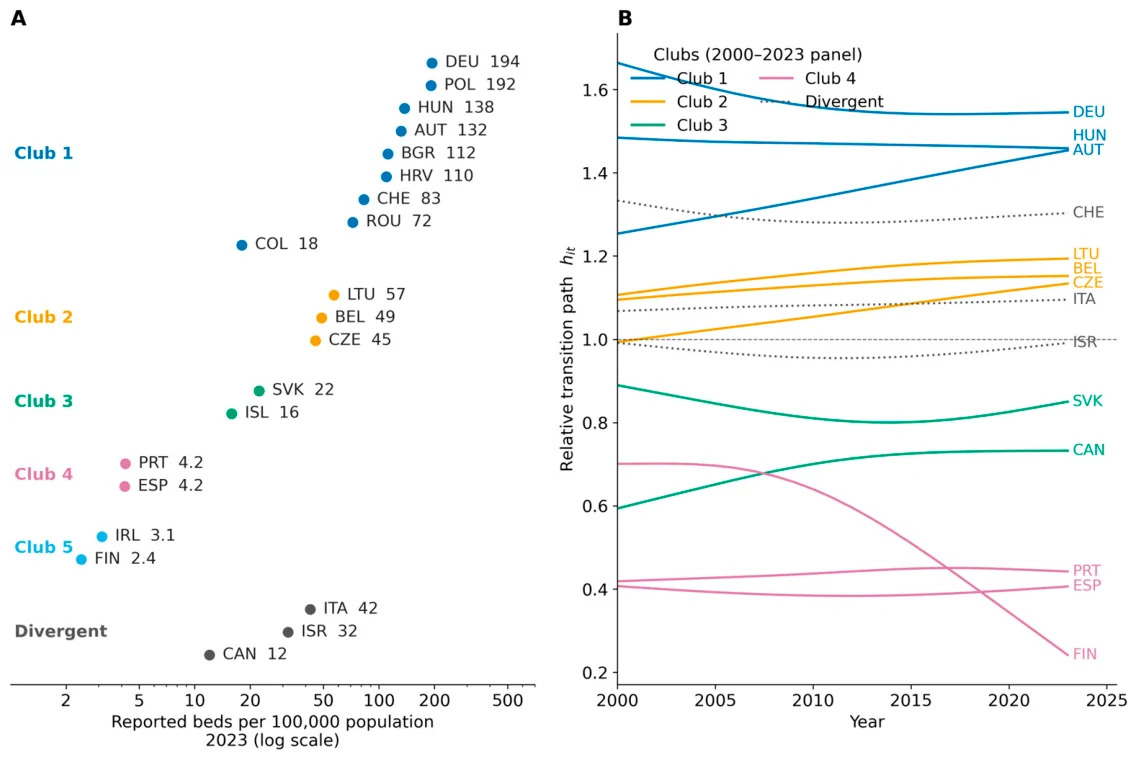
Exploratory convergence clubs and relative transition paths.

Exploratory clustering identified several groups of trajectories alongside ungrouped countries (Table 6; Figure 3). Membership differs between the 24-year balanced core and the 14-year extended core, and should not be read as a fixed classification of national rehabilitation systems. The corrected algorithm tests all countries outside a candidate core during the sieve step. Table A1 reports dimension-specific recovery performance, which is lower for shorter panels; Table A2 shows the corresponding older-population-denominator classifications.

Figure 3A shows the corrected exploratory club assignments for the extended core, positioned at reported 2023 bed capacity. Figure 3B shows relative transition paths *h_it_* for the balanced core, calculated from HP-smoothed log series relative to their cross-country mean. Club labels refer to the panel analysed and are not interchangeable between panels. Simulation results in Table A1 describe the uncertainty of short-panel classification.

Capacity growth showed no consistent positive association with population ageing (Table 7). In the combined panel, the mean-group coefficient was +0.2587 (95% CI −0.1309 to +0.6484), the CCEMG coefficient +0.1333 (−0.6679 to +0.9345), and the Driscoll–Kraay estimate −0.0471 (−0.0961 to +0.0020). All primary intervals included zero. One distributed-lag variant yielded a negative coefficient of −0.0611 (*p* = 0.033; Table A5); this result was not consistent across estimators. The long-difference correlation was −0.156 (*p* = 0.594). These results describe uncertain and specification-dependent demographic associations rather than proof that rehabilitation capacity is unrelated to need.

Specification checks continued to reject panel-wide log-t convergence and did not reveal consistent positive demographic associations (Table 8). The pre-COVID-19, rehabilitation-share, and older-population-denominator analyses address different features of the observed panel. Positive HAC dispersion trends should be interpreted alongside the country-bootstrap intervals, and the negative distributed-lag association remains visible in Table A5. These checks do not establish that reporting differences or community substitution are absent.

## 4. Discussion

Reported inpatient rehabilitation-bed stocks remained markedly heterogeneous across the included countries. The panel-wide convergence tests and imprecise catch-up estimates provide little evidence that these trajectories moved toward a common level. Observed dispersion rose in the balanced core, although country-resampling uncertainty qualifies a general claim of increasing divergence. Growth in the older-population share did not show a consistent positive association with capacity growth. Together, the results describe one component of rehabilitation infrastructure without equating its distribution with the adequacy of rehabilitation provision.

Differences in delivery models are central to interpreting inpatient indicators. Seijas and colleagues documented variation in the availability and integration of inpatient, outpatient, community, home-based, remote, and eldercare rehabilitation [10]. Winitsky and colleagues showed that financing and service-delivery arrangements also vary across countries [11]. The German health-system review describes a distinct institutional context [34], while the cited Finnish review notes that rehabilitation is not always reported separately and describes its delivery within health-center inpatient facilities [35]. These sources support interpreting bed stocks within national service structures, rather than attributing the cross-country range to differences in total rehabilitation access.

Reporting conventions can affect trajectories as well as levels. Eurostat identifies differences in national coverage and organization and notes that changes in collection can produce series breaks [15]. WHO’s guidance on rehabilitative-care expenditure similarly emphasizes the importance of consistent functional classification, although it concerns expenditure rather than bed counts [36]. Expressing rehabilitation beds as a share of all hospital beds or comparing within-country changes does not eliminate these issues. The convergent feature of our sensitivity analyses is the absence of panel-wide convergence in the reported series, not proof that the series measure identical service packages.

A lower inpatient bed stock can coexist with substantial rehabilitation outside hospitals. Langhorne and colleagues’ review of early supported discharge after stroke supports selected community-based pathways [37]. The telerehabilitation reviews by Laver and colleagues and Suso-Martí and colleagues concern specific populations and interventions and cannot establish the adequacy of a national service mix [38,39]. Lam and colleagues’ account of spinal-cord-injury care illustrates a continuum incorporating residential, community day, and respite services [40]. These examples explain why inpatient bed comparisons need complementary information on service use, staffing, and outcomes.

The demographic models answer a narrower question than whether planning follows need. Age is relevant to functioning and service demand [8,9], but neither the share aged 80+ years nor the share aged 65+ measures rehabilitation-eligible episodes, disability severity or access. Jesus and colleagues examined rehabilitation workforce supply against condition-based need estimates [41,42], whereas the present study relates one inpatient resource to population ageing. The studies therefore are concerned with related planning questions but different exposures and resources. Our imprecise and sign-varying estimates leave room for associations that cannot be resolved by this panel and do not identify a causal role for institutional history.

The findings extend the health-resource convergence literature to a reported functional bed category. Hitiris and Nixon, Panopoulou and Pantelidis, and Albulescu examined convergence in health expenditure [21,22,23], while Harikrishnan and Hiremath considered health-center provision [24]. Expenditure, facilities, and designated rehabilitation beds respond to different financing and organizational processes; so, agreement in the existence of heterogeneous trajectories does not imply a common mechanism. In this paper, dimension-matched simulations additionally show why rejection of overall convergence should be distinguished from confidence in individual short-panel club assignments.

For planners, the practical implication is to combine compatible indicators before inferring a capacity gap. Bed stocks and their age denominators can be monitored alongside the rehabilitation workforce, outpatient and community activity, waiting times, and functioning outcomes. This approach is consistent with Rehabilitation 2030 [5], the workforce challenges described by Jesus and colleagues [43], and the service perspectives of the *European rehabilitation White Book* [44]. It permits country-specific decisions about inpatient and community capacity without treating more inpatient beds as an automatic indicator of better provision.

Future work could link consistent functional bed series with rehabilitation expenditure, workforce, and service-use data [11,36,41,43]. Evaluations around financing reforms or changes in delivery models could then test mechanisms that this ecological panel cannot identify. The documented disruption of rehabilitation during COVID-19 [45] also supports examining institutional and service changes directly, rather than inferring them from aggregate trajectories alone.

The study’s strengths are its long observation window, transparent source combination, observed balanced panels, and multiple approaches to convergence and demographic association. Reproduction from archived data and dimension-matched simulation checks make the empirical results and their operating conditions inspectable.

The included country set is selective, reflecting incomplete reporting and the original extraction mapping. In particular, Moldova, North Macedonia, and Serbia had positive rehabilitation-bed observations in the Eurostat archive but were not included because they fell outside the original country mapping (Supplementary Table S1). Their inclusion could have altered the combined-panel estimates and, where their series were sufficiently complete, the composition of the extended core. The analytical sample therefore does not provide complete coverage of Europe or the OECD, and the results apply to the analyzed series rather than to all European or OECD health systems. Differences in classification and service organization constrain comparisons of both levels and changes. Country-bootstrap intervals show substantial country-composition uncertainty in dispersion trends. Short time series limit the stability of club assignments, and simulation performance is specific to the chosen data-generating processes rather than a probability that empirical membership is correct. Finally, demographic age shares are incomplete need proxies and the ecological models cannot determine whether national rehabilitation planning is adequate or which mechanisms produced the observed trajectories.

## 5. Conclusions

Reported inpatient rehabilitation-bed capacity showed persistent cross-country differences and no detected panel-wide convergence in the 27 included European and OECD countries during 2000–2023, a sample that does not provide complete coverage of either group. Population ageing was not consistently positively associated with capacity growth. The findings support harmonized monitoring of this inpatient resource alongside community rehabilitation, workforce, service use, and functioning outcomes; they do not establish an absence of need-based planning.

## Figures and Tables

**Table 1:** Descriptive statistics, union panel (27 countries, 2000–2023, 504 country-years).

Variable	N	Mean	SD	Min	Median	Max
Rehabilitation beds per 100,000 population	504	55.65	56.20	2.42	39.83	228.65
Rehabilitation beds per 1000 population aged 80+	504	13.24	12.66	0.41	9.88	63.51
Rehabilitation beds per 1000 population aged 65+	504	3.21	3.05	0.10	2.38	14.11
Rehabilitation share of total hospital beds (%)	504	9.45	7.72	0.32	7.59	30.57
Population aged 80+ (%)	504	4.22	1.24	1.05	4.10	7.60
Population aged 65+ (%)	504	16.82	3.49	5.84	17.11	24.00
GDP per capita (PPP, constant intl USD)	504	47,785	20,741	14,037	46,236	136,772

Data: Eurostat and World Bank for population structure; World Bank for GDP. A total of 426 of 504 capacity cells are Eurostat-sourced, while 78 are OECD-sourced.

**Table 2:** Panel diagnostics: cross-sectional dependence and unit roots.

Series (Panel)	CD ^1^ Statistic	CD ^1^ *p*	Mean Abs. Pairwise rho	CIPS Level	CIPS First Diff.	Order
ln rehab beds per 100k (union, 27)	7.66	<0.001	0.498	-	-	-
ln rehab beds per 100k (balanced, 14)	7.84	<0.001	0.520	−1.88	−3.44 *	I(1)
ln rehab beds per 100k aged 80+ (balanced)	11.93	<0.001	0.585	−1.83	−2.94 *	I(1)
Population 80+ share (balanced)	45.14	<0.001	0.966	−1.64	−2.54 *	I(1)
Delta ln rehab beds (union)	0.97	0.331	0.253	-	-	-
Delta ln rehab beds (balanced)	1.81	0.071	0.191	-	-	-

* indicates rejection of the unit-root null at 5%. Pesaran–Yamagata slope-homogeneity test for the responsiveness specification: Delta = 1.09 (*p* = 0.274), adjusted Delta = 1.20 (*p* = 0.230)—homogeneity not rejected. ^1^ CD = Pesaran CD test (H0: cross-sectional independence). CIPS 5% critical value = −2.39 (intercept, one lag).

**Table 3:** Sigma-convergence: cross-sectional dispersion of log capacity.

Outcome (Panel)	N	SD (First Year)	SD (Last Year)	Change [Bootstrap 95% CI]	Trend Per Decade (HAC *p*)
ln beds per 100k, balanced core	14	1.256 (2000)	1.387 (2023)	+0.130 [−0.156, +0.423]	+0.082 (*p* < 0.001)
ln beds per 100k aged 80+, balanced core	14	1.273 (2000)	1.425 (2023)	+0.153 [−0.162, +0.468]	+0.102 (*p* < 0.001)
ln beds per 100k aged 65+, balanced core	14	1.249 (2000)	1.423 (2023)	+0.174 [−0.121, +0.505]	+0.104 (*p* < 0.001)
ln beds per 100k, extended core 2010–2023	21	1.362 (2010)	1.382 (2023)	+0.020 [−0.259, +0.298]	+0.028 (*p* = 0.145)
ln beds per 100k aged 80+, extended core	21	1.254 (2010)	1.383 (2023)	+0.130 [−0.103, +0.388]	+0.112 (*p* < 0.001)

Bootstrap: 2000 country-cluster resamples. Trend: OLS of SD_t on year, Newey–West (3 lags) standard errors.

**Table 4:** Beta-convergence: growth-on-initial-level regressions.

Specification	Beta	SE ^2^	*p*	95% CI	Obs.	Countries
Union, 5 yr windows, unconditional	−0.0042	0.0052	0.423	[−0.0144, +0.0061]	103	26
Union, 5 yr windows, conditional ^1^	−0.0043	0.0047	0.354	[−0.0134, +0.0048]	103	26
Balanced core, 5 yr windows, unconditional	+0.0016	0.0052	0.762	[−0.0086, +0.0117]	70	14
Balanced core, 5 yr windows, conditional	+0.0020	0.0054	0.710	[−0.0085, +0.0125]	70	14
Union, per older population (per 80+), unconditional	−0.0008	0.0043	0.860	[−0.0092, +0.0077]	103	26
Cross-Section 2000–2023, balanced core	+0.0019	0.0046	0.680	[−0.0072, +0.0110]	14	14

^1^ The union-panel conditional model includes the annualized change in the population share aged 80 years and older (coefficient = −0.070; *p*= 0.318) and initial ln GDP per capita (coefficient = −0.0304; *p*= 0.026). ^2^ Window fixed effects included; SEs clustered by country (cross-section: HC1).

**Table 5:** Phillips–Sul log-t convergence test.

Panel/Outcome	N	T	log-t b	SE	t-Statistic	Convergence (5%) ^1^
Balanced core 2000–2023, raw capacity	14	24	−0.999	0.021	−47.26	rejected
Balanced core 2000–2023, per older population (per 80+)	14	24	−1.159	0.023	−51.03	rejected
Extended core 2010–2023, raw capacity	21	14	−1.003	0.014	−70.42	rejected
Extended core 2010–2023, per older population	21	14	−1.168	0.008	−141.15	rejected

^1^ One-sided 5% critical value −1.65 (Phillips and Sul 2007) [19]; HP-smoothed (lambda = 400) log series; first 30% of the sample discarded in the log-t regression; HAC standard errors.

**Table 6:** Convergence clubs. (**a**) Extended Core (21 Countries, 2010–2023); (**b**) Balanced Core (14 Countries, 2000–2023).

(a)				
Club	Members	log-t b (SE)	*t*	Mean Beds/100,000 in 2023
Club 1	AUT, BGR, CHE, COL, DEU, HRV, HUN, POL, ROU	+0.060 (0.095)	+0.63	116.5
Club 2	BEL, CZE, LTU	+0.288 (0.133)	+2.17	50.3
Club 3	ISL, SVK	+2.147 (1.428)	+1.50	19.1
Club 4	ESP, PRT	+0.720 (0.323)	+2.23	4.2
Club 5	FIN, IRL	+0.718 (1.549)	+0.46	2.8
Ungrouped	CAN, ISR, ITA	-	-	28.8
**(b)**				
**Club**	**Members**	**log-t b (SE)**	***t***	**Mean Beds/100,000 in 2023**
Club 1	AUT, DEU, HUN	+1.128 (0.101)	+11.11	154.4
Club 2	BEL, CZE, LTU	+0.451 (0.180)	+2.51	50.3
Club 3	CAN, SVK	−0.010 (0.400)	−0.02	17.1
Club 4	ESP, FIN, PRT	+0.862 (0.825)	+1.05	3.6
Ungrouped	CHE, ISR, ITA	-	-	52.3

Note: A club is retained when its log-t t-statistic exceeds −1.65. Mean capacity from the union panel, 2023.

**Table 7:** Responsiveness: capacity growth on lagged ageing growth.

Estimator (Panel)	Coefficient	SE	*p*	95% CI	N
Mean Group (union)	+0.2587	0.1988	0.193	[−0.1309, +0.6484]	24
CCEMG (union)	+0.1333	0.4088	0.744	[−0.6679, +0.9345]	26
Within-FE, Driscoll–Kraay (union)	−0.0471	0.0250	0.060	[−0.0961, +0.0020]	450
Mean Group (balanced core)	+0.0527	0.1168	0.652	[−0.1763, +0.2817]	14
CCEMG (balanced core)	−0.1622	0.1549	0.295	[−0.4659, +0.1415]	14
Within-FE, Driscoll–Kraay (balanced core)	−0.0301	0.0404	0.456	[−0.1092, +0.0490]	308
Contemporaneous ageing growth, MG (union)	+0.2493	0.1496	0.096	[−0.0439, +0.5425]	26
65+ share variant, DK (union)	−0.0343	0.0271	0.207	[−0.0875, +0.0189]	450

Note: Dependent variable: Delta ln rehab beds. N is countries for mean-group estimators and observations for pooled estimators. Long-difference cross-section (2000–2023, balanced core): Pearson r = −0.156 (*p* = 0.594); OLS slope −0.080 (*p* = 0.284), N = 14.

**Table 8:** Robustness matrix.

Check	Sigma Trend/Decade (*p*)	Beta (*p*)	log-t t
Headline (2000–2023)	+0.082 (*p* < 0.001)	−0.0042 (*p* = 0.423)	−47.26
COVID-19-era excluded (2000–2019)	+0.064 (*p* < 0.001)	−0.0033 (*p* = 0.629)	−124.74
Rehab share of total beds	+0.048 (*p* < 0.001)	−0.0053 (*p* = 0.238)	−54.88
per older population per 65+	+0.104 (*p* < 0.001)	−0.0011 (*p* = 0.820)	−33.30
Sub-period 2000–2011 (beta)	-	−0.0029 (*p* = 0.610)	-
Sub-period 2012–2023 (beta)	-	−0.0046 (*p* = 0.498)	-
Leave-one-country-out range	-	[−0.0075, −0.0004]	[−54.58, −30.18]

## Data Availability

All data are publicly available: Eurostat (https://ec.europa.eu/eurostat), the OECD Health Statistics data explorer (https://data-explorer.oecd.org), and the World Bank Open Data API (https://data.worldbank.org). The full reproducible pipeline—Python and R code, every raw API response, the assembled panels and all outputs—is provided with the submission and will be deposited in a public repository upon acceptance.

## Supplementary Materials

The following supporting information can be downloaded at: https://www.mdpi.com/article/10.3390/healthcare14203500/s1, Table S1: Country coverage and inclusion in the archived-source analysis; Table S2: Joint country-bootstrap uncertainty for dispersion trends and endpoint changes; Table S3: Dimension-matched Monte Carlo design and detailed operating performance.

## Abbreviations

The following abbreviations are used in this manuscript:

CCEMG	Common Correlated Effects Mean Group
CD	Cross-Sectional Dependence (Test)
CI	Confidence Interval
CIPS	Cross-Sectionally Augmented Im–Pesaran–Shin (Test)
CSD	Cross-Sectional Dependence
EU	European Union
GBD	Global Burden of Disease
GDP	Gross Domestic Product
HAC	Heteroskedasticity and Autocorrelation-Consistent
HC.2	Rehabilitative Care (SHA 2011 Function)
HP	Hodrick–Prescott (Filter)
MG	Mean Group
OECD	Organisation For Economic Co-Operation And Development
OLS	Ordinary Least Squares
PPP	Purchasing Power Parity
SD	Standard Deviation
SE	Standard Error
SHA	System of Health Accounts
WHO	World Health Organization

## Nomenclature

The following nonstandard statistical terms are used in this manuscript:

**Term**	**Definition**
Ecological (macro-panel) study	A design in which the unit of analysis is the country-year rather than the individual patient. Associations observed at country level need not hold for individuals (the ecological fallacy).
Union panel	The full working dataset: all 27 countries with at least one observation (504 country-years, 2000–2023), used for the regression-based analyses, which tolerate an unbalanced panel.
Balanced core	The 14 countries observed in every year 2000–2023 with no interpolated cells. Used for convergence statistics, which require a constant set of countries over time.
Extended core	The 21 countries observed in every year 2010–2023 with no interpolated cells; a wider country set over a shorter window, used as a complement to the balanced core.
Population shares (aged 80+, 65+)	The proportions aged 80 years or older and 65 years or older. They describe population ageing but are not complete measures of rehabilitation need.
Capacity per older population	Reported inpatient rehabilitation beds divided by the population aged 80+ or 65+. This changes the demographic denominator without measuring disability or service requirements.
Sigma-convergence (σ)	A sustained fall over time in the dispersion of a variable across countries; a rising dispersion series indicates divergence.
Cross-sectional standard deviation of log capacity	The standard deviation, computed across countries within a single year, of log capacity, either raw (beds per 100,000 population) or per older population (per 100,000 aged 80+). Its time path is what defines sigma-convergence or sigma-divergence.
Beta-convergence (β)	The tendency of countries starting from a lower initial level to grow faster, so that laggards catch up. Estimated by regressing subsequent growth on the initial log level; a negative coefficient indicates convergence.
Panel-wide beta-convergence	Beta-convergence estimated for all countries jointly, i.e., one common catch-up coefficient, as opposed to convergence occurring only within subgroups of countries.
Convergence half-life	The time a beta-convergence estimate implies would be needed to close half of the existing gap between countries; a very long half-life means catch-up is negligible in practice.
Convergence club	A subgroup of countries that converge towards a common long-run level or path while remaining separated from other subgroups, so that the panel as a whole does not converge.
Phillips-Sul log-t test	A regression-based test of convergence: the quantity log(H1/Ht) − 2 log(log t), formed from the cross-sectional variance of relative transition paths, is regressed on log t. A one-sided t-statistic below −1.65 rejects convergence for the group tested.
log-t coefficient b	The slope of the log-t regression. Its sign and one-sided test concern convergence in the group analysed; uncertainty and short-panel operating characteristics must be considered.
Relative transition path	Here, each country’s HP-smoothed log-capacity series divided by the cross-country mean of that smoothed log series in the same year.
Club-clustering algorithm (sieve)	Countries are ordered by their mean over the final half of the series. A core is selected, all remaining countries are considered for inclusion, and the remainder is tested before recursive clustering. Adjacent qualifying clubs may be merged.
Hodrick-Prescott (HP) filter, smoothing parameter λ	A filter separating a series into trend and cyclical components; λ governs the degree of smoothing (λ = 400 here) and is applied to the log series before the log-t test.
Trimming fraction (r)	The proportion of the initial observations discarded before the log-t regression (r = 0.3 here), because early periods are dominated by transitional dynamics.
Finite-T limitation	A loss of precision that arises when the number of time periods (T) is small: units still in mid-transition can be assigned to the wrong club; so, borderline memberships should be interpreted cautiously.
Monte-Carlo validation (size and power)	Assessment of an estimator or test on many simulated datasets whose true structure is known, applied here to the whole estimator suite. Size is the rejection rate when the null hypothesis is true; power is the rejection rate when it is false.
Transitional heterogeneity	Country-specific, temporary differences in the path towards a common long-run outcome. The log-t test is designed to remain valid in its presence, unlike tests based on the levels of the series.
Cross-sectional dependence (CSD)	Correlation of country series or regression residuals within the same year, typically caused by shared shocks or common policy. If ignored, it invalidates conventional panel inference.
Pesaran CD test	A diagnostic test for cross-sectional dependence. The null hypothesis is cross-sectional independence; rejection indicates that countries’ series are driven in part by common factors.
Mean |ρ|	The average absolute pairwise correlation between countries’ series, reported alongside the CD statistic as a measure of how strong that dependence is.
CIPS panel unit-root test	The cross-sectionally augmented Im-Pesaran-Shin test of whether panel series contain a unit root, allowing for cross-sectional dependence. The null is a unit root in all series; the 5% critical value used here is −2.39.
Unit root/I(1) series	A series is I(1), or contains a unit root, when its level wanders without returning to a fixed mean, so that shocks are permanent and differencing is required before regression.
Pesaran-Yamagata slope-homogeneity test (Delta, adjusted Delta)	A test of whether the regression slope is the same in every country, reported as a Delta statistic and a small-sample adjusted Delta. Rejection implies country-specific slopes; a non-rejection, as obtained here, means slope homogeneity cannot be ruled out.
Mean group (MG) estimator	An estimator that fits the regression separately for each country and averages the resulting coefficients, allowing every country its own slope. Pesaran–Smith standard errors are computed from the dispersion of those country-specific coefficients.
CCEMG estimator	Common Correlated Effects Mean Group: a mean-group estimator that augments each country regression with cross-sectional averages of the variables in order to purge unobserved common factors; used here as the dependence-robust check.
Pooled within fixed-effects estimator	A single regression estimated on deviations from each country’s own mean, so that time-invariant country characteristics are absorbed and one common slope is imposed on all countries.
Window fixed effects	Indicator variables for each growth window (2000–2005, 2005–2010, 2010–2015, 2015–2020, 2020–2023), absorbing shocks common to all countries within that period.
Distributed lag	A specification that includes both the current and one or more lagged values of an explanatory variable, allowing the response to accumulate over several years.
Long-difference cross-section	A single regression estimated on the total change between the first and the last year of the panel, with one observation per country; used to capture very slow-moving relationships.
HAC standard errors	Heteroskedasticity- and autocorrelation-consistent standard errors: the general family to which the Newey–West and Driscoll–Kraay estimators below belong.
Newey–West (3-lag) standard errors	HAC standard errors for a time-series regression; “3-lag” is the number of lags over which autocorrelation is allowed for.
Driscoll–Kraay standard errors	Panel standard errors that remain valid under autocorrelation, heteroskedasticity, and cross-sectional dependence simultaneously.
Cluster-robust standard errors	Standard errors that allow observations of the same country to be correlated with one another, i.e., clustering by country; used for the panel growth regressions.
HC1	A separate, heteroskedasticity-robust covariance estimator with a finite-sample correction. It is used for the long-run cross-sectional regression, which has one observation per country and therefore nothing to cluster on.
Country-cluster bootstrap	Resampling complete country trajectories. Original pointwise and endpoint intervals use 2000 resamples; the additional joint analysis of slopes and endpoint differences uses 5000.
Leave-one-country-out deletion	Repeating an analysis after omitting each country in turn to assess influence. Stability to this check does not remove other uncertainty or measurement limitations.

## Appendix A

This appendix reports the supporting material behind the main analysis. Table A1 reports simulation performance at the actual panel dimensions. Table A2 and Table A3 present older-population-denominator club assignments and specification sensitivity; Table A4 reports the original independent R comparison; Table A5 retains the demographic-association variants. Supplementary Tables S1–S3 provide country eligibility, joint uncertainty estimates, and full simulation specifications.

**Table A1:** Dimension-matched Monte Carlo operating performance of the corrected Phillips–Sul implementation. (**a**) Percentages with 95% Wilson Intervals; 2000 Replications Per Scenario and Panel Dimension; (**b**) Original Diagnostic and Estimator Checks Under Their Specified Data-Generating Processes.

(a)					
Countries × Years	Converging Panel: Rejection	Two Clubs: Rejection	Two Clubs: Exact Recovery	Divergent Panel: Rejection	Dispersed Initial Conditions: Exact Recovery
14 × 24	0.00 [0.00, 0.19]	100.00 [99.81, 100.00]	99.95 [99.72, 99.99]	100.00 [99.81, 100.00]	93.75 [92.60, 94.73]
21 × 14	0.00 [0.00, 0.19]	100.00 [99.81, 100.00]	90.40 [89.03, 91.61]	100.00 [99.81, 100.00]	51.15 [48.96, 53.34]
14 × 20	0.00 [0.00, 0.19]	100.00 [99.81, 100.00]	98.15 [97.46, 98.65]	100.00 [99.81, 100.00]	80.80 [79.02, 82.47]
**(b)**					
**Routine**		**Known Truth**		**Recovered**	
Pesaran CD		independent panel		CD = −0.73, *p* = 0.46 (no rejection)	
Pesaran CD		common-factor panel		CD = 93.5, *p* < 0.001	
CIPS		random walk (I(1))		−1.53 vs. cv −2.26: unit root not rejected	
CIPS		stationary AR(0.3)		−2.58: unit root rejected	
Pesaran–Yamagata		homogeneous slopes		Δ*_adj_* = −1.61, *p* = 0.107 (no rejection)	
Pesaran–Yamagata		heterogeneous slopes		Δ*_adj_* = 256.6, *p* < 0.001	
CCEMG		β = −0.50 (common factor)		−0.500	
CS-ARDL		β = −0.50		−0.510	
AMG		β = −0.50		−0.500	
MG (CSD-naïve)		β = −0.50		−0.102 (biased, as expected)	

Each simulation generated log-level panels with prespecified convergence or two-club structure and used the same smoothing and test settings as the empirical analysis. The displayed rejection rates are scenario-specific and do not estimate general accuracy or calibrate the full convergence-null boundary. Exact recovery requires both inferred clubs to match their known members, regardless of label order. Supplementary Table S3 specifies the processes, seeds and Monte Carlo uncertainty.

**Table A2:** Exploratory clubs for capacity per population aged 80+. (**a**) Balanced Core 2000–2023: log-t b = −1.159 (SE 0.023), *t* = −51.03; (**b**) Extended Core 2010–2023: log-t b = −1.168 (SE 0.008), t = −141.15.

(a)			
Club	Members	log-t b (SE)	t
Club 1	AUT, CHE, DEU, HUN	+0.189 (0.051)	+3.74
Club 2	BEL, CZE, ISR, LTU	+0.752 (0.099)	+7.57
Club 3	ITA, SVK	+0.047 (1.068)	+0.04
Club 4	ESP, FIN, PRT	+2.160 (0.882)	+2.45
Ungrouped	CAN	-	-
**(b)**			
**Club**	**Members**	**log-t b (SE)**	**t**
Club 1	AUT, BGR, CHE, COL, DEU, HRV, HUN, ROU	+0.662 (0.134)	+4.95
Club 2	CZE, ISR, LTU	+0.697 (0.120)	+5.81
Club 3	ISL, ITA, SVK	+1.222 (0.225)	+5.42
Club 4	ESP, PRT	+3.839 (0.859)	+4.47
Ungrouped	BEL, CAN, FIN, IRL, POL	-	-

**Table A3:** Phillips–Sul sensitivity (balanced core, raw capacity).

Setting	log-t b	t
Trimming r = 0.2	−0.990	−79.04
Trimming r = 0.3 (headline)	−0.999	−47.26
Trimming r = 0.4	−1.027	−44.38
HP λ = 100	−1.035	−42.72
HP λ = 400 (headline)	−0.999	−47.26
HP λ = 1600	−0.954	−95.13
No smoothing	−1.050	−36.24

Convergence is rejected (t < −1.65) under every setting.

**Table A4:** R cross-validation of headline results.

Quantity	Python (Pipeline)	R (Independent Recomputation)	Verdict
log-t slope b, balanced core, raw	−0.999	−0.9990	identical (4 dp)
log-t t-statistic, raw	−47.26	−44.39	same inference; difference = HAC small-sample correction
log-t slope b, per older population	−1.159	−1.1586	identical (3 dp)
log-t t-statistic, per older population	−51.03	−47.94	same inference
beta (union, unconditional)	−0.0042	−0.0041857	identical (4 dp)
beta SE (clustered)	0.0052	0.0052277	identical
beta *p*	0.423	0.4253	same inference; *p*-values differ slightly
Pesaran CD, ln beds (balanced)	7.84	7.84	identical
mean abs. pairwise rho	0.520	0.520	identical
CIPS level/first difference	−1.88/−3.44	−1.881/−3.4357	identical
sigma trend per decade	+0.0825	+0.0825	identical

R 4.6.0 with plm, sandwich, lmtest. The R script recomputes the HP filter, transition paths, and the log-t regression.

**Table A5:** Responsiveness variants (union panel).

Variant	Coefficient	SE	*p*
Contemporaneous ageing growth, MG	+0.2493	0.1496	0.096
Contemporaneous ageing growth, DK-FE	+0.0463	0.0494	0.349
65+ share (lagged), MG	−0.2415	0.2533	0.341
65+ share (lagged), DK-FE	−0.0343	0.0271	0.207
Distributed lag, DK-FE: Δs80(t)	+0.0448	0.0456	0.326
Distributed lag, DK-FE: Δs80(t − 1)	−0.0611	0.0286	0.033

The distributed-lag coefficient for the prior-year change in the 80+ share was negative (*p* = 0.033). Its inconsistency with the other specifications does not justify either dismissing it as noise or interpreting it as evidence of inverse need-based planning.

## References

[1] Cieza A., Causey K., Kamenov K., Hanson S.W., Chatterji S., Vos T. (2020). Global estimates of the need for rehabilitation based on the Global Burden of Disease study 2019: A systematic analysis for the Global Burden of Disease Study 2019. Lancet.

[2] Ahmad D., Alemu Y.M., Bagheri N., Burns R.A., Talukder A. (2024). Global incidence, prevalence, years lived with disability (YLDs), disability-adjusted life-years (DALYs), and healthy life expectancy (HALE) for 371 diseases and injuries in 204 countries and territories and 811 subnational locations, 1990–2021: A systematic analysis for the Global Burden of Disease Study 2021. Lancet.

[3] Steinmetz J.D., Seeher K.M., Schiess N., Nichols E., Cao B., Servili C., Cavallera V., Cousin E., Hagins H., Moberg M.E. (2024). Global, regional, and national burden of disorders affecting the nervous system, 1990–2021: A systematic analysis for the Global Burden of Disease Study 2021. Lancet Neurol..

[4] Ferreira M.L., de Luca K., Haile L.M., Steinmetz J.D., Culbreth G.T., Cross M., A Kopec J., Ferreira P.H., Blyth F.M., Buchbinder R. (2023). Global, regional, and national burden of low back pain, 1990–2020, its attributable risk factors, and projections to 2050: A systematic analysis of the Global Burden of Disease Study 2021. Lancet Rheumatol..

[5] Gimigliano F., Negrini S. (2017). The World Health Organization “Rehabilitation 2030: A call for action”. Eur. J. Phys. Rehabil. Med..

[6] Stucki G., Bickenbach J., Gutenbrunner C., Melvin J. (2018). Rehabilitation: The health strategy of the 21st century. J. Rehabil. Med..

[7] Kamenov K., Mills J.A., Chatterji S., Cieza A. (2019). Needs and unmet needs for rehabilitation services: A scoping review. Disabil. Rehabil..

[8] Beard J.R., Officer A., de Carvalho I.A., Sadana R., Pot A.M., Michel J.P., Lloyd-Sherlock P., Epping-Jordan J.E., Peeters G.M.E., Mahanani W.R. (2016). The World report on ageing and health: A policy framework for healthy ageing. Lancet.

[9] de Meijer C., Wouterse B., Polder J., Koopmanschap M. (2013). The effect of population aging on health expenditure growth: A critical review. Eur. J. Ageing.

[10] Seijas V., Maritz R., Morsch P., Kleinitz P., Morgan C., Yee J., Moreira B., Hodel J., Mishra S., Sabariego C. (2025). International stakeholder consultation on models of rehabilitation service delivery to foster healthy ageing: Results of a cross-sectional survey involving 124 countries and all World Health Organization regions. Eur. J. Phys. Rehabil. Med..

[11] Winitsky S., Webb E., Polin K., Shuftan N., Waitzberg R. (2025). Strengthening Rehabilitation Care: Learning from Countries’ Experiences on Organizing Service Delivery and Financing.

[12] Ravaghi H., Alidoost S., Mannion R., Bélorgeot V.D. (2020). Models and methods for determining the optimal number of beds in hospitals and regions: A systematic scoping review. BMC Health Serv. Res..

[13] Ettelt S., Nolte E., Thomson S., Mays N. (2008). Capacity Planning in Health Care: A Review of the International Experience.

[14] OECD (2023). Health at a Glance 2023: OECD Indicators.

[15] Eurostat (2024). Healthcare Resource Statistics—Beds (dataset hlth_rs_bds1, Joint Questionnaire on Non-Monetary Health Care Statistics).

[16] OECD, Eurostat, World Health Organization (2017). A System of Health Accounts 2011: Revised Edition.

[17] Barro R.J., Sala-i-Martin X. (1992). Convergence. J. Polit. Econ..

[18] Quah D.T. (1996). Twin peaks: Growth and convergence in models of distribution dynamics. Econ. J..

[19] Phillips P.C.B., Sul D. (2007). Transition modeling and econometric convergence tests. Econometrica.

[20] Phillips P.C.B., Sul D. (2009). Economic transition and growth. J. Appl. Econom..

[21] Hitiris T., Nixon J. (2001). Convergence of health care expenditure in the EU countries. Appl. Econ. Lett..

[22] Panopoulou E., Pantelidis T. (2012). Convergence in per capita health expenditures and health outcomes in the OECD countries. Appl. Econ..

[23] Albulescu C.T. (2022). Health care expenditure in the European Union countries: New insights about the convergence process. Int. J. Environ. Res. Public Health.

[24] Harikrishnan K.S., Hiremath G.S. (2024). Inequality in Public Provision of Healthcare: Do Fiscal Transfers Matter?. J. Public Aff..

[25] OECD (2026). Hospital Beds by Function of Health Care (Dataset DSD_HEALTH_REAC_HOSP@DF_BEDS_FUNC).

[26] World Bank (2026). World Development Indicators and Health Nutrition and Population Statistics (SP.POP.80UP.FE, SP.POP.80UP.MA, SP.POP.TOTL, SP.POP.65UP.TO.ZS, NY.GDP.PCAP.PP.KD).

[27] Pesaran M.H. (2021). General diagnostic tests for cross-sectional dependence in panels. Empir. Econ..

[28] Pesaran M.H. (2007). A simple panel unit root test in the presence of cross-section dependence. J. Appl. Econom..

[29] Pesaran M.H., Yamagata T. (2008). Testing slope homogeneity in large panels. J. Econom..

[30] Hodrick R.J., Prescott E.C. (1997). Postwar US business cycles: An empirical investigation. J. Money Credit Bank..

[31] Pesaran M.H., Smith R. (1995). Estimating long-run relationships from dynamic heterogeneous panels. J. Econom..

[32] Pesaran M.H. (2006). Estimation and inference in large heterogeneous panels with a multifactor error structure. Econometrica.

[33] Driscoll J.C., Kraay A.C. (1998). Consistent covariance matrix estimation with spatially dependent panel data. Rev. Econ. Stat..

[34] Busse R., Blümel M. (2014). Germany: Health system review. Health Syst. Transit..

[35] Keskimäki I., Tynkkynen L.K., Reissell E., Koivusalo M., Syrjä V., Vuorenkoski L., Rechel B., Karanikolos M. (2019). Finland: Health system review. Health Syst. Transit..

[36] World Health Organization (2026). Tracking Rehabilitative Care Expenditure Under the System of Health Accounts 2011.

[37] Langhorne P., Baylan S., Early Supported Discharge Trialists (2017). Early supported discharge services for people with acute stroke. Cochrane Database Syst. Rev..

[38] Laver K.E., Adey-Wakeling Z., Crotty M., Lannin N.A., George S., Sherrington C. (2020). Telerehabilitation services for stroke. Cochrane Database Syst. Rev..

[39] Suso-Martí L., La Touche R., Herranz-Gómez A., Angulo-Díaz-Parreño S., Paris-Alemany A., Cuenca-Martínez F. (2021). Effectiveness of telerehabilitation in physical therapist practice: An umbrella and mapping review with meta-meta-analysis. Phys. Ther..

[40] Lam C.Y., Su I.Y.W., Law J.Y.M. (2024). Transitional Care for Spinal Cord Injuries in Hong Kong SAR, China: A Narrative Review of the Local Experience. Healthcare.

[41] Jesus T.S., Landry M.D., Hoenig H., Dussault G., Koh G.C., Fronteira I. (2022). Is physical rehabilitation need associated with the rehabilitation workforce supply? An ecological study across 35 high-income countries. Int. J. Health Policy Manag..

[42] Jesus T.S., Landry M.D., Brooks D., Hoenig H. (2020). Physical rehabilitation needs per condition type: Results from the Global Burden of Disease study 2017. Arch. Phys. Med. Rehabil..

[43] Jesus T.S., Landry M.D., Dussault G., Fronteira I. (2017). Human resources for health (and rehabilitation): Six rehab-workforce challenges for the century. Hum. Resour. Health.

[44] European Physical and Rehabilitation Medicine Bodies Alliance (2018). White Book on Physical and Rehabilitation Medicine in Europe. Introductions, Executive Summary, and Methodology. Eur. J. Phys. Rehabil. Med..

[45] Negrini S., Grabljevec K., Boldrini P., Kiekens C., Moslavac S., Zampolini M., Christodoulou N. (2020). Up to 2.2 million people experiencing disability suffer collateral damage each day of COVID-19 lockdown in Europe. Eur. J. Phys. Rehabil. Med..

