# Supplementary material for: Persistent Differences in Reported Inpatient Rehabilitation-Bed Capacity Across 27 European and OECD Countries
Source: Healthcare (Basel). 2026 Oct 18;14(20):3500. doi: 10.3390/healthcare14203500 (PMC13704588; doi:10.3390/healthcare14203500)
Supplement: Supplementary file 1 [file healthcare-14-03500-s001.zip › healthcare-4602928-supplementary.pdf]

Supplementary material

Persistent Differences in Reported Inpatient Rehabilitation-Bed Capacity across 27 European and OECD Countries

Table S1. Country coverage and inclusion in the archived-source analysis

The retained analytic panel comprises 27 countries and 504 country-years. The archived sources contain 56 country labels after aggregate groups are removed. Fifty countries fall within the original extraction mapping: 27 were included and 23 had no target rehabilitation-bed observations in 2000–2023. Six additional Eurostat countries were outside that mapping. This table reports observed availability; absence of a target series does not establish zero beds or explain why a country did not report.

S1a. Countries included in the combined panel

Country	Observed period	Years	Eurostat / OECD observations	Core panels
Austria (AUT)	2000–2023	24	24 / 0	2000–2023; 2010–2023
Belgium (BEL)	2000–2023	24	24 / 0	2000–2023; 2010–2023
Bulgaria (BGR)	2005–2023	19	19 / 0	2010–2023
Canada (CAN)	2000–2023	24	0 / 24	2000–2023; 2010–2023
Switzerland (CHE)	2000–2023	24	24 / 0	2000–2023; 2010–2023
Colombia (COL)	2010–2023	14	0 / 14	2010–2023
Czechia (CZE)	2000–2023	24	24 / 0	2000–2023; 2010–2023
Germany (DEU)	2000–2023	24	24 / 0	2000–2023; 2010–2023
Spain (ESP)	2000–2023	24	24 / 0	2000–2023; 2010–2023
Finland (FIN)	2000–2023	24	24 / 0	2000–2023; 2010–2023
Greece (GRC)	2015–2023	9	9 / 0	Combined only
Croatia (HRV)	2009–2023	15	15 / 0	2010–2023
Hungary (HUN)	2000–2023	24	24 / 0	2000–2023; 2010–2023
Ireland (IRL)	2009–2023	15	15 / 0	2010–2023
Iceland (ISL)	2007–2023	17	17 / 0	2010–2023
Israel (ISR)	2000–2023	24	0 / 24	2000–2023; 2010–2023
Italy (ITA)	2000–2023	24	24 / 0	2000–2023; 2010–2023
Korea (KOR)	2014–2023	10	0 / 10	Combined only
Lithuania (LTU)	2000–2023	24	24 / 0	2000–2023; 2010–2023
Luxembourg (LUX)	2013–2023	11	11 / 0	Combined only
Malta (MLT)	2014–2023	10	10 / 0	Combined only
Netherlands (NLD)	2015–2023	9	9 / 0	Combined only
Poland (POL)	2010–2023	14	14 / 0	2010–2023
Portugal (PRT)	2000–2023	24	24 / 0	2000–2023; 2010–2023
Romania (ROU)	2005–2023	19	19 / 0	2010–2023
Slovakia (SVK)	2000–2023	24	24 / 0	2000–2023; 2010–2023
United States (USA)	2017–2022	6	0 / 6	Combined only

The 2000–2023 core contains 14 countries and the 2010–2023 core 21. The construction rules required observed endpoints, at least 22 or 12 observations respectively, and no gap longer than two years. All retained core series were complete; no interpolated values were used. Source counts identify the selected source after preferring Eurostat over overlapping OECD observations.

S1b. Archived-source countries excluded from the combined panel

Country	Within original mapping	Years with positive target data	Exclusion reason
Albania (ALB)	No	0	Outside original country mapping
Argentina (ARG)	Yes	0	No target observation in original source scope
Australia (AUS)	Yes	0	No target observation in original source scope
Brazil (BRA)	Yes	0	No target observation in original source scope
Chile (CHL)	Yes	0	No target observation in original source scope
China (People's Republic of) (CHN)	Yes	0	No target observation in original source scope
Costa Rica (CRI)	Yes	0	No target observation in original source scope
Cyprus (CYP)	Yes	0	No target observation in original source scope
Denmark (DNK)	Yes	0	No target observation in original source scope
Estonia (EST)	Yes	0	No target observation in original source scope
France (FRA)	Yes	0	No target observation in original source scope
United Kingdom (GBR)	Yes	0	No target observation in original source scope
Indonesia (IDN)	Yes	0	No target observation in original source scope
India (IND)	Yes	0	No target observation in original source scope
Japan (JPN)	Yes	0	No target observation in original source scope
Liechtenstein (LIE)	No	0	Outside original country mapping; available target values were zero
Latvia (LVA)	Yes	0	No target observation in original source scope
Moldova (MDA)	No	9	Outside original country mapping
Mexico (MEX)	Yes	0	No target observation in original source scope
North Macedonia (MKD)	No	8	Outside original country mapping
Montenegro (MNE)	No	0	Outside original country mapping
Norway (NOR)	Yes	0	No target observation in original source scope
New Zealand (NZL)	Yes	0	No target observation in original source scope
Russia (RUS)	Yes	0	No target observation in original source scope
Serbia (SRB)	No	18	Outside original country mapping
Slovenia (SVN)	Yes	0	No target observation in original source scope
Sweden (SWE)	Yes	0	No target observation in original source scope
Türkiye (TUR)	Yes	0	No target observation in original source scope
South Africa (ZAF)	Yes	0	No target observation in original source scope

Positive-data counts for countries outside the mapping describe availability in the stored source extracts, not an added analytic sample. Moldova, North Macedonia and Serbia had positive Eurostat target observations but were not in the original mapping. The study does not claim complete geographic representation.

Table S2. Joint country-bootstrap uncertainty for dispersion trends and endpoint changes

For each specification, 5,000 resamples selected complete country trajectories with replacement (seed 20260930). The linear slope and final-minus-initial difference were recomputed together in each resample. Percentile intervals therefore describe sensitivity to country composition. HAC intervals describe the temporal slope conditional on the observed countries. The original 2,000-resample endpoint intervals remain in main Table 3; their small numerical differences from this table reflect different bootstrap draws.

Panel and denominator	Endpoint change [country 95% CI]	Trend per decade [country 95% CI]	HAC 95% CI for trend	HAC p
14 countries; total population	+0.130<br>[-0.152, +0.428]	+0.082<br>[-0.047, +0.243]	[+0.053, +0.112]	<0.001
14 countries; population 80+	+0.153<br>[-0.167, +0.452]	+0.102<br>[-0.049, +0.259]	[+0.063, +0.141]	<0.001
14 countries; population 65+	+0.174<br>[-0.128, +0.495]	+0.104<br>[-0.032, +0.275]	[+0.060, +0.147]	<0.001
21 countries; total population	+0.020<br>[-0.264, +0.299]	+0.028<br>[-0.201, +0.258]	[-0.010, +0.067]	0.145
21 countries; population 80+	+0.130<br>[-0.106, +0.391]	+0.112<br>[-0.070, +0.327]	[+0.094, +0.131]	<0.001

The two core periods are 2000–2023 and 2010–2023. All country-bootstrap intervals in this table include zero. These are resampling intervals for the selected countries; they do not make the non-random country sample representative of Europe or the OECD.

Table S3. Dimension-matched Monte Carlo design and detailed operating performance

All scenarios generated log-level input panels $Y_{it} = \delta_{it}\mu_t$. The common component was μ_t = 3 + 0.03t plus cumulative independent normal increments with standard deviation 0.01. The same HP smoothing (λ = 400), 30% trimming, Bartlett HAC settings and corrected club-selection algorithm were applied as in the empirical analysis. A total of 2,000 replications was run for each scenario and each panel dimension. Independent R code reproduced every corrected empirical club assignment.

Scenario A: One limiting loading of 1. Initial deviations were normal with standard deviation 0.20 and decayed as exp(-0.20t); independent normal noise with standard deviation 0.20 was additionally scaled by exp(-0.20t)/log(t+1).

Scenario B: The same transition process, with limiting loadings 1.3 and 0.7. Club sizes were 7/7 for N = 14 and 10/11 for N = 21.

Scenario C: Loadings followed 1 plus cumulative independent normal increments with standard deviation 0.08.

Scenario D: The two-club process with initial-deviation standard deviation increased to 0.35, probing slower transitions from more dispersed starting positions. Generated log values were retained without censoring.

Countries × years	Scenario	Overall rejection % [95% CI]	MCSE percentage points	Exact recovery % [95% CI]	MCSE percentage points
14 × 24	A	0/2000<br>0.00<br>[0.00, 0.19]	0.000	-	-
14 × 24	B	2000/2000<br>100.00<br>[99.81, 100.00]	0.000	1999/2000<br>99.95<br>[99.72, 99.99]	0.050
14 × 24	C	2000/2000<br>100.00<br>[99.81, 100.00]	0.000	-	-
14 × 24	D	2000/2000<br>100.00<br>[99.81, 100.00]	0.000	1875/2000<br>93.75<br>[92.60, 94.73]	0.541
21 × 14	A	0/2000<br>0.00<br>[0.00, 0.19]	0.000	-	-
21 × 14	B	2000/2000<br>100.00<br>[99.81, 100.00]	0.000	1808/2000<br>90.40<br>[89.03, 91.61]	0.659
21 × 14	C	2000/2000<br>100.00<br>[99.81, 100.00]	0.000	-	-
21 × 14	D	1998/2000<br>99.90<br>[99.64, 99.97]	0.071	1023/2000<br>51.15<br>[48.96, 53.34]	1.118
14 × 20	A	0/2000<br>0.00<br>[0.00, 0.19]	0.000	-	-
14 × 20	B	2000/2000<br>100.00<br>[99.81, 100.00]	0.000	1963/2000<br>98.15<br>[97.46, 98.65]	0.301
14 × 20	C	2000/2000<br>100.00<br>[99.81, 100.00]	0.000	-	-
14 × 20	D	2000/2000<br>100.00<br>[99.81, 100.00]	0.000	1616/2000<br>80.80<br>[79.02, 82.47]	0.881

Rejection uses $t < -1.65$. Exact recovery requires exactly two inferred clubs with memberships identical to the two true clubs, ignoring label order. Intervals are Wilson binomial intervals; $MCSE(p) = \sqrt{\frac{p(1-p)}{2{,}000}}$

A zero plug-in MCSE at a boundary proportion does not imply perfect accuracy: its binomial interval remains non-degenerate. The master seed was 20260930, with independent scenario/dimension streams defined by the master seed, N, T and the ASCII scenario code. These scenarios assess operating characteristics of the implementation; they do not calibrate every point on the convergence-null boundary or assign probabilities to empirical country memberships.
